# Ameliorative effects of Schisandrin B on *Schistosoma mansoni*-induced hepatic fibrosis in vivo

**DOI:** 10.1371/journal.pntd.0009554

**Published:** 2021-06-23

**Authors:** Ho Yin Pekkle Lam, Ting-Ruei Liang, Shih-Yi Peng

**Affiliations:** 1 Institute of Medical Sciences, Tzu Chi University, Hualien, Taiwan; 2 Department of Biochemistry, School of Medicine, Tzu Chi University, Hualien, Taiwan; 3 Ph.D. Program in Pharmacology and Toxicology, School of Medicine, Tzu Chi University, Hualien, Taiwan; James Cook University Division of Tropical Health and Medicine, AUSTRALIA

## Abstract

Schistosomiasis is second only to malaria as the most devastating parasitic disease in the world. It is caused by the helminths *Schistosoma mansoni* (*S*. *mansoni*), *S*. *haematobium*, or *S*. *japonicum*. Typically, patients with schistosomiasis suffer from symptoms of liver fibrosis and hepatosplenomegaly. Currently, patients were treated with praziquantel. Although praziquantel effectively kills the worm, it cannot prevent re-infection or resolve liver fibrosis. Also, current treatment options are not ample to completely cure liver fibrosis and splenic damages. Moreover, resistance of praziquantel has been reported in vivo and in vitro studies. Therefore, finding new effective treatment agents is urgently needed. Schisandrin B (Sch B) of *Schisandra chinensis* has been shown to protect against different liver injuries including fatty liver disease, hepatotoxicity, fibrosis, and hepatoma. We herein investigate the potential of using Sch B to treat *S*. *mansoni*-induced liver fibrosis. Results from the present study demonstrate that Sch B is beneficial in treating *S*. *mansoni*-induced liver fibrosis and splenic damages, through inhibition of inflammasome activation and apoptosis; and aside from that regulates host immune responses. Besides, Sch B treatment damages male adult worm in the mice, consequently helps to reduce egg production and lessen the parasite burden.

## Author summary

Patients infected with *Schistosoma mansoni* (*S*. *mansoni*) inevitably results in liver fibrosis and hepatosplenomegaly. Currently, praziquantel is the drug of choice for these patients. Although praziquantel effectively kills the worm, it cannot prevent re-infection or resolve liver fibrosis. Moreover, resistance of praziquantel has been suggested in vivo and in vitro studies. Therefore, finding new and effective treatment options is urgently needed. Schisandrin B (Sch B) of *Schisandra chinensis* has been shown to protect against different liver injuries including fatty liver disease, hepatotoxicity, fibrosis, and hepatoma. In this study we have investigated the potential of using Sch B to treat *S*. *mansoni*-induced liver fibrosis.

## Introduction

Schistosomiasis is a disease caused by the helminths *Schistosoma mansoni* (*S*. *mansoni*), *S*. *haematobium*, or *S*. *japonicum*. Infectious cercariae penetrate human hosts and develop into an adult worm which resides in the mesenteric venules. The majority of the eggs produced by the adult worm are not excreted and are lodged in the liver of hosts. Eventually, this drives a granulomatous response against helminths’ eggs, and, alternatively, causes liver fibrosis [1]. Granuloma is caused by cytokine-mediated endocytosis/phagocytosis [2]. Also, a large number of immune cells including lymphocytes, eosinophils, and macrophages are responsible for the host’s immune responses as they produce certain inflammatory cytokines such as interleukins (IL) -1β, IL-18, TNF-α, and T helper 2 (Th2) cytokines such as IL-4 and IL-5 [2,3].

Inflammasomes have been found to regulate important aspects of tissue inflammation. The activation of NOD-like receptor family pyrin domain-containing three (NLRP3) inflammasomes has been shown during *Schistosoma*-infected liver fibrosis [4–6]. Once NLRP3 is activated, it recruits an adaptor protein, apoptosis-associated speck-like protein containing a caspase recruitment domain (ASC), and induces activation of caspase-1. Activated caspase-1 then processes pro-IL-1β and pro-IL-18 to its mature, active form. In addition, activated caspase-1 cleaves gasdermin D (GSDMD) into two domains. The cleaved N-terminal fragment of GSDMD forms pores in the cell membrane to cause pyroptosis, a type of inflammatory-related programmed cell death [7,8].

Moreover, the activation of inflammasomes is involved in the apoptotic pathway. NLRP3 has been shown to activate both apoptosis and pyroptosis when the cell encounter a bacterial antigen [9,10]. Another inflammasome, absent in melanoma 2 (AIM2), has also been shown to activate apoptotic caspases in parallel with inflammatory caspase [9,11]. Currently, two apoptotic signaling cascades, the extrinsic and intrinsic pathways, have been distinguished. When a cell is stimulated by an extracellular antigen, the Fas plasma membrane death receptor is activated to form a death complex which recruits a FAS-associating death domain-containing protein (FADD) and caspase-8. Caspase-8 then activates caspase-3 and processes apoptosis [12]. On the other hand, when a cell is stimulated by an intracellular signal, the mitochondria release cytochrome c into the cytosol. Cytochrome c then triggers downstream caspase-9 and processes caspases-3 [12,13]. Both extrinsic and intrinsic pathway stimulates downstream caspases-3, which is the penultimate enzyme for the execution of the apoptotic process [14]. Apoptosis has also been shown to governs the elimination of *S*. *japonicum* in hosts [15].

Praziquantel is currently the drug of choice for schistosomiasis. Although praziquantel effectively kills the worm, it cannot prevent re-infection or resolve liver fibrosis. Moreover, resistance of praziquantel has been suggested in vivo and in vitro studies [16–19]. Therefore, finding new and effective agents to treat schistosomiasis is urgently needed. Currently, many new natural products have revealed surprisingly effective antiparasitic properties [20]. In addition, many well-known drugs that are used in the clinical setting have their origins in nature. For example, quinine, isolated from the cinchona (Quina Quina) tree for treating *Plasmodium* infection, has been followed by the subsequent development of chloroquine, primaquine, and mefloquine [21].

*Schisandra chinensis*, among many natural herbs, has been shown to protect against different liver injuries including alcoholic fatty liver disease [22], non-alcohol induced fatty liver disease [23], chemical-induced liver toxicity, fibrosis [24,25], and hepatocellular carcinoma [26]. Several studies have reported that Schisandrin B (Sch B) of *S*. *chinensis* displayed a potent anti-inflammatory and anti-oxidant effect in different disease models [27–32]. Previously it was also reported from our laboratory that Sch B is beneficial in treating *Angiostrongylus cantonensis* infection by downregulating infection-induced inflammation and apoptosis [33]. We herein investigate the potential of using Sch B to treat *S*. *mansoni*-induced liver fibrosis.

## Results

### Schisandrin B ameliorates *S*. *mansoni*-induced liver fibrosis

**Fig 1:**
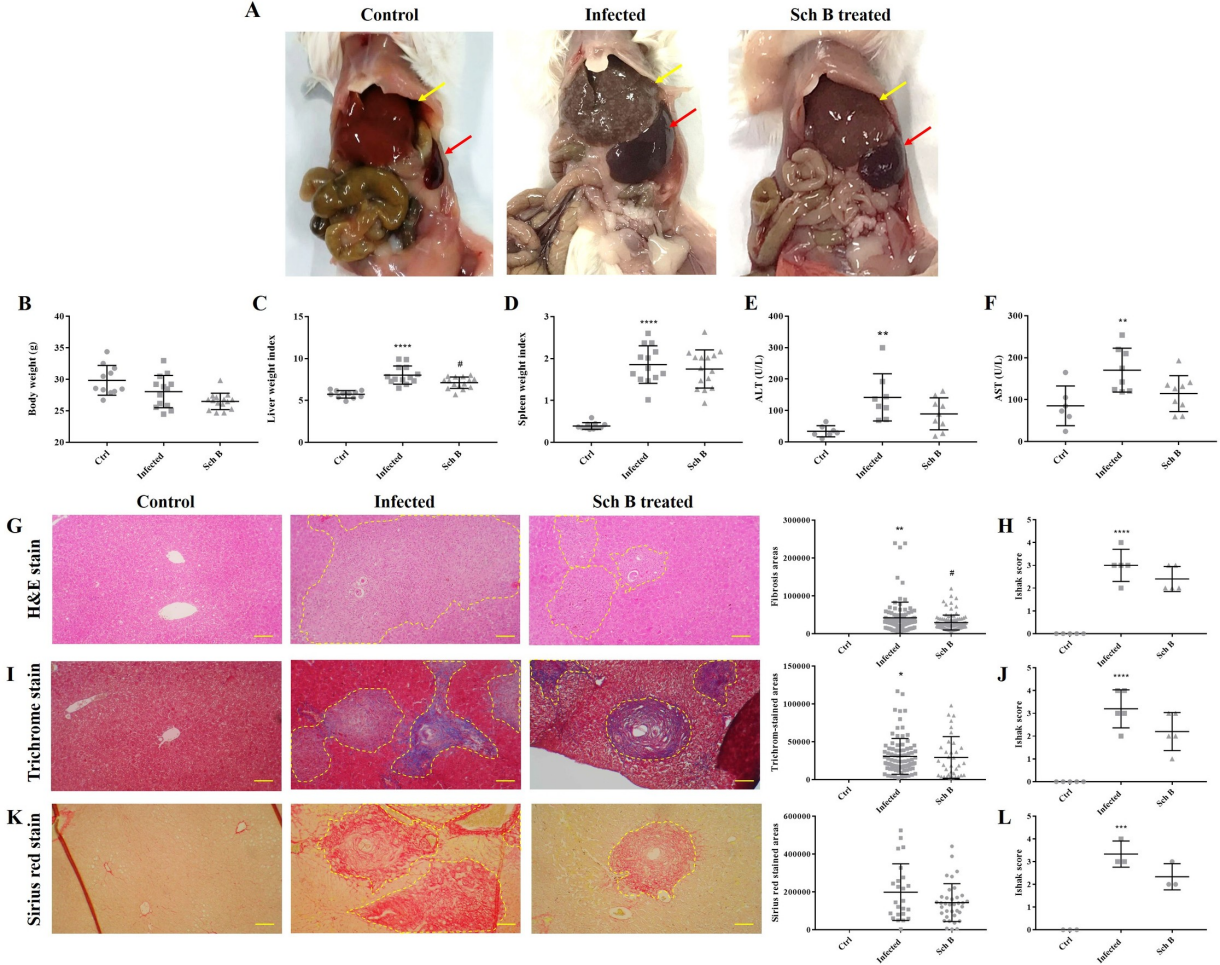
Sch B treatment improves liver function and ameliorates liver fibrosis in *S*. *mansoni*-infected mice. (A) Representative images showing gross pathology of control, infected, or Sch B treated mice. Hepatosplenomegaly was observed in infected mice (liver, yellow arrows; spleen, red arrows). White spots seen on the surface of liver indicate granuloma nodules. Effect of Sch B on (B) body weight changes, (C) liver weight index (calculated by liver weight relative to body weight), and (D) spleen weight index (calculated by spleen weight relative to body weight). All results are shown as mean ± S.D. (n = 11–15). (E) ALT and (F) AST levels measured in the serum. Hemolyzed samples were excluded. All results are shown as mean ± S.D. (n = 6–9). (G) Representative images showing H&E staining of liver sections of the mice. Fibrotic areas were encircled by a yellow dotted line. (H) Fibrosis was evaluated by Ishak fibrosis scoring based on H&E staining. (I) Representative images of Masson’s trichrome staining on liver sections of the mice. Collagen were stained as blue which was encircled by a yellow dotted line. (J) Ishak fibrosis scoring based on Masson’s trichrome staining. (K) Representative images of Sirius red staining on liver sections of the mice. Collagen were stained as red and were encircled by a yellow dotted line. (L) Ishak fibrosis scoring based on Sirius red staining. For quantification in (G, I, and K), the number and the corresponding areas of each circled area were potted directly on the graph. Each dot represents one fibrotic or positively stained area. Quantification or scoring was performed on five (H&E staining and Masson’s trichrome staining) or three (Sirius red staining) slides in each group. 15 random microscopic fields were examined on each slide. Images are shown at 100× magnifications and scale bars correspond to 200 μm. * *P*-value < 0.05, ** *P*-value < 0.01, *** *P*-value < 0.001, and **** *P*-value < 0.0001 compared with control group; # *P*-value < 0.05 compared with infected group.

Infection of *S*. *mansoni* for 8 weeks resulted in severe hepatosplenomegaly in mice, along with the formation of granuloma nodules on the external surface of livers (Fig 1A). However, treatment with Sch B ameliorated the mouse’s hepatosplenomegaly and pathological nodules on livers (Fig 1A). In addition, serum level of ALT and AST decreased after *S*. *mansoni*-infected mice treated with Sch B (Fig 1E and 1F). Histological analysis of the control mice showed normal architecture of the liver with central veins and portal tracts. However, eggs were found trapped in the liver in *S*. *mansoni*-infected mice and were surrounded by a marked increase of inflammatory infiltrates, forming fibrotic tissues (Fig 1G). Masson’s trichrome and Sirius red staining exhibit a high amount of collagen deposition around the trapped eggs (Fig 1I and 1K). Upon treatment with Sch B, the number and size of the fibrotic tissues were reduced (Fig 1G, 1I and 1K). The severity of fibrosis was also evaluated by the Ishak fibrosis scoring system, which showed similar beneficial effects of Sch B on *S*. *mansoni*-induced fibrosis (Fig 1H, 1J and 1L).

### Schisandrin B alleviates *S*. *mansoni*-induced liver fibrosis by inhibiting HSC activation

**Fig 2:**
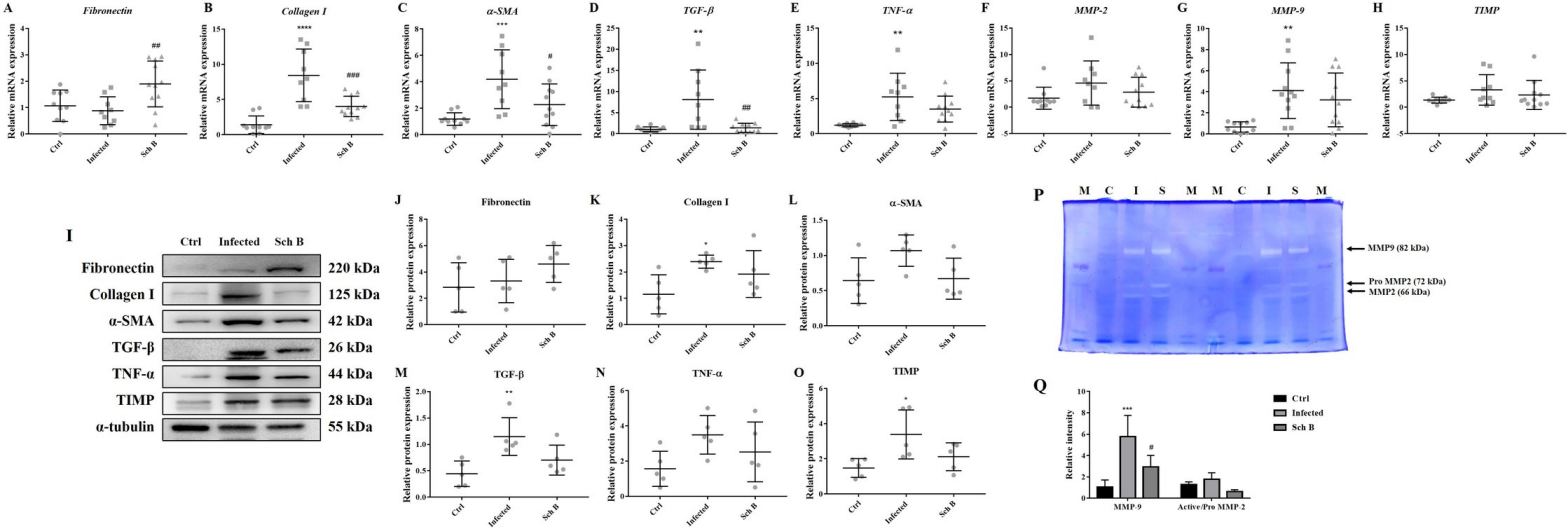
Sch B downregulates fibrotic genes and protein expression in *S*. *mansoni*-infected mice. (A-H) RNA transcription levels of fibrotic markers including *fibronectin*, *collagen I*, *α-SMA*, *TGF-β*, *TNF-α*, *MMP-2*, *MMP-9*, and *TIMP*, measured by qPCR. Data are presented as mean ± S.D. (n = 9–11). (I) Representative western blot images showing protein levels of fibrotic markers. (J-O) Protein expression levels of fibrotic markers, relative to that of α-tubulin. Results are presented as mean ± S.D. (n = 5). (P) MMP-2 and MMP-9 activities were detected by gelatin zymography. The activities were detected as unstained bands on a blue background. M, markers; C, control group; I, infected group; S, Sch B-treated group. Two independent experiments were shown. (Q) The bands were quantified by densitometry using image J. Results are presented as mean ± S.D. (n = 3). * *P*-value < 0.05, ** *P*-value < 0.01, *** *P*-value < 0.001, and **** *P*-value < 0.0001 compared with control group; # *P*-value < 0.05, and ### *P*-value < 0.001 compared with infected group.

Activation of hepatic stellate cells (HSCs) to differentiate into myofibroblast-like phenotype is a major hallmark of liver fibrosis [34]. Although countless transient changes in gene transcription occur during HSCs activation, the major transcriptional targets include fibronectin, collagen I, α-SMA, TGF-β, TNF-α, MMP-2, MMP-9, and TIMP [35]. We, therefore, investigate the above markers in their mRNA and protein levels. Our results showed that expression of collagen I, α-SMA, TGF-β, TNF-α, and TIMP, as well as MMP-2 and MMP-9 activities, increased in livers of infected mice, which decrease upon treatment of Sch B (Fig 2B–2H and 2K–2Q). Unexpectedly, the expression of one fibrosis-related marker, fibronectin, although slightly increased during infection, did not decrease after treatment. Quite the contrary, the expression of fibronectin further increased after Sch B is administered (Fig 2A and 2J).

### Schisandrin B reduces *S*. *mansoni*-induced inflammation through inhibition of inflammasome

**Fig 3:**
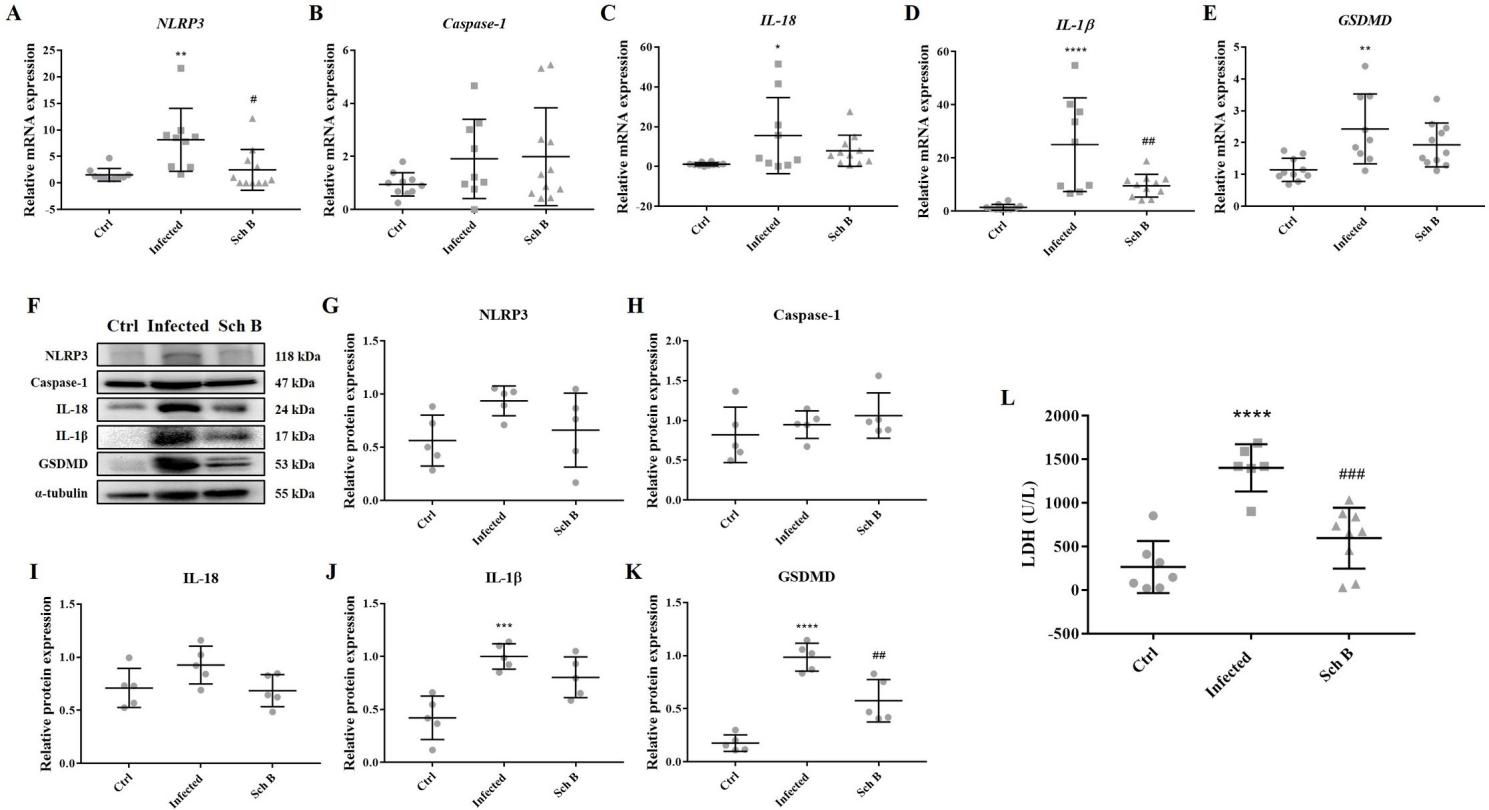
Sch B ameliorates inflammasome activation and inhibits pyroptosis in *S*. *mansoni*-infected mice. (A-E) RNA transcription levels of inflammasomes markers, measured by qPCR. Data are presented as mean ± S.D. (n = 9–11). (F) Representative western blot images showing protein levels of inflammasomes markers. (G-K) Protein expression levels of inflammasomes markers, relative to that of α-tubulin. Results are presented as mean ± S.D. (n = 5). (L) Serum level of lactate dehydrogenase (LDH). Hemolyzed samples were excluded. Results are presented as mean ± S.D. (n = 6–9). * *P*-value < 0.05, ** *P*-value < 0.01, *** *P*-value < 0.001, and **** *P*-value < 0.0001 compared with control group; # *P*-value < 0.05, ## *P*-value < 0.01, and ### *P*-value < 0.001 compared with infected group.

Inflammation plays an important role in the development and progression of liver fibrosis [36,37]. Previously, *S*. *mansoni* infection has been shown to activates NLRP3 inflammasomes in vitro and in vivo models [4–6]. Since Sch B has been shown to ameliorates inflammasome activation in various inflammatory models [27–29,33], to also verify the protective effects of Sch B against *S*. *mansoni*-induced liver fibrosis, we examined the effects of Sch B on the NLRP3 inflammasome pathway. Our results showed that RNA and protein levels of NLRP3 inflammasome, activated caspase-1, IL-18, and IL-1β were significantly increased in the livers of *S*. *mansoni*-infected mice (Fig 3A–3D and 3F–3J). The increase in these inflammasome components was reduced following Sch B treatment. Moreover, Sch B reduced the expression of pyroptotic markers GSDMD and concentration of serum LDH (Fig 3E, 3K and 3L). Taken together, these data suggested Sch B can reduce *S*. *mansoni*-induced inflammation by inhibiting activation of NLRP3 inflammasome and pyroptosis, thereby displaying a potential role to inhibit *S*. *mansoni*-induced liver fibrosis.

### Schisandrin B reduces macrophages proliferation and their activation of the inflammasome

**Fig 4:**
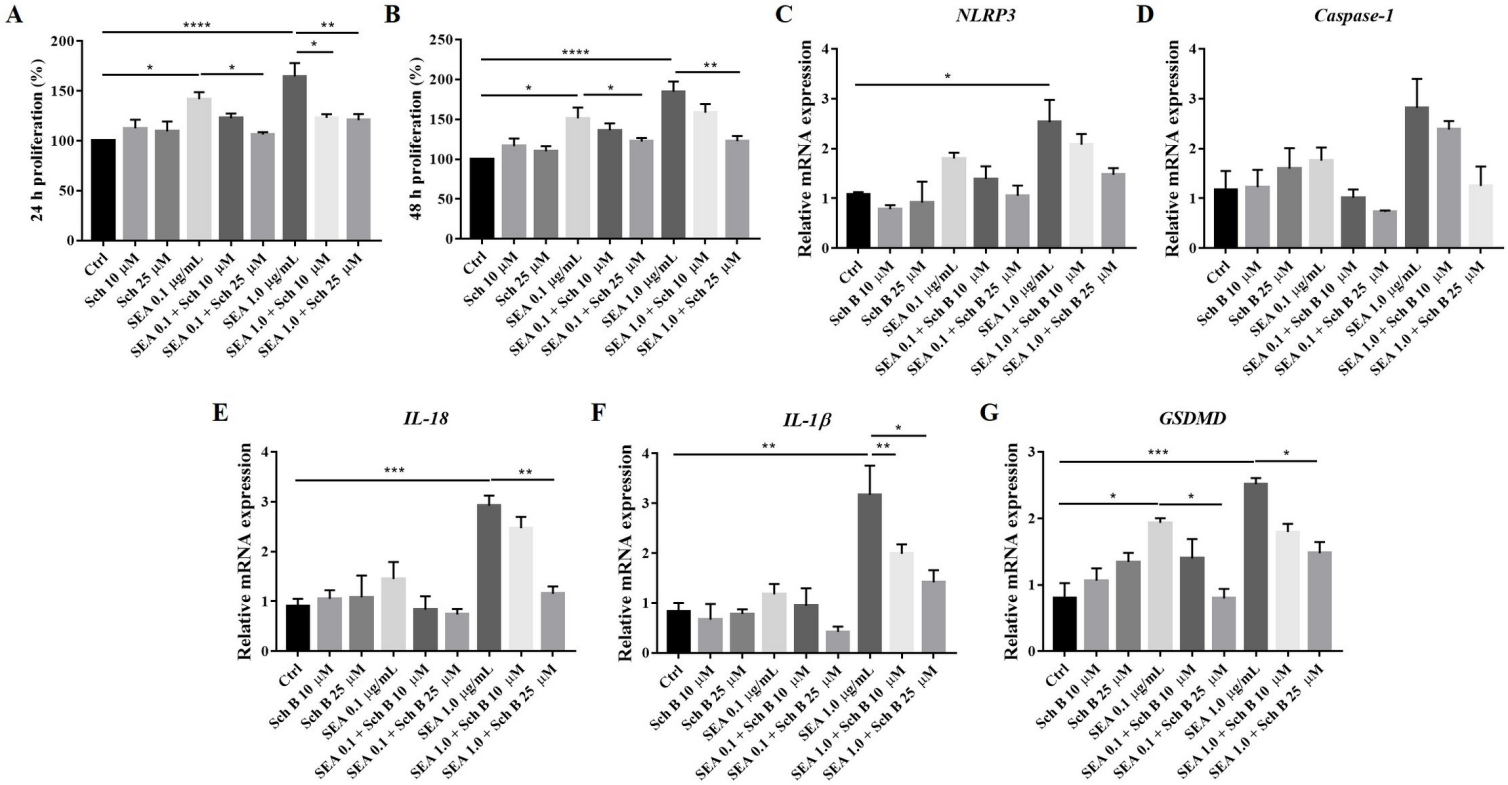
Proliferation and inflammasome activation in RAW264.7 cells stimulated by *S*. *mansoni* soluble egg antigens. RAW264.7 cells were treated with indicated concentration of soluble egg antigen (SEA) and Sch B. (A-B) 24- and 48- hour proliferation of RAW264.7 cells, measured by WST-1 assay. (C-G) mRNA expression of inflammasome component. All results are presented as the mean ± S.D. from three independent experiments. * *P*-value < 0.05, ** *P*-value < 0.01, *** *P*-value < 0.001, and **** *P*-value < 0.0001 compared between groups.

Inflammasomes are presented in various immune cells [38] and have been shown to express in macrophages during schistosomiasis [39,40]. To further clarify how Sch B affects inflammasome, we perform in vitro study using soluble egg antigen (SEA)-stimulated RAW 264.7 cells. Proliferation of RAW264.7 macrophages was significantly increased by 0.1 μg/mL and 1.0 μg/mL SEA stimulation; while the use of 10 μM and 25 μM Sch B decreased their proliferation (Fig 4A and 4B). This was further confirmed by immunohistochemistry staining, which suggests a significant decrease of macrophages by Sch B treatment, but not other immune cells (S1 Fig). In addition, mRNA expression of NLRP3 inflammasome components was increased by SEA stimulation, which was also decreased by Sch B treatment (Fig 4C–4G). Thus, we suggest that Sch B decreased *S*. *mansoni*-induced macrophage proliferation along with inflammasome activation.

### Schisandrin B suppresses *S*. *mansoni*-induced liver cell apoptosis

**Fig 5:**
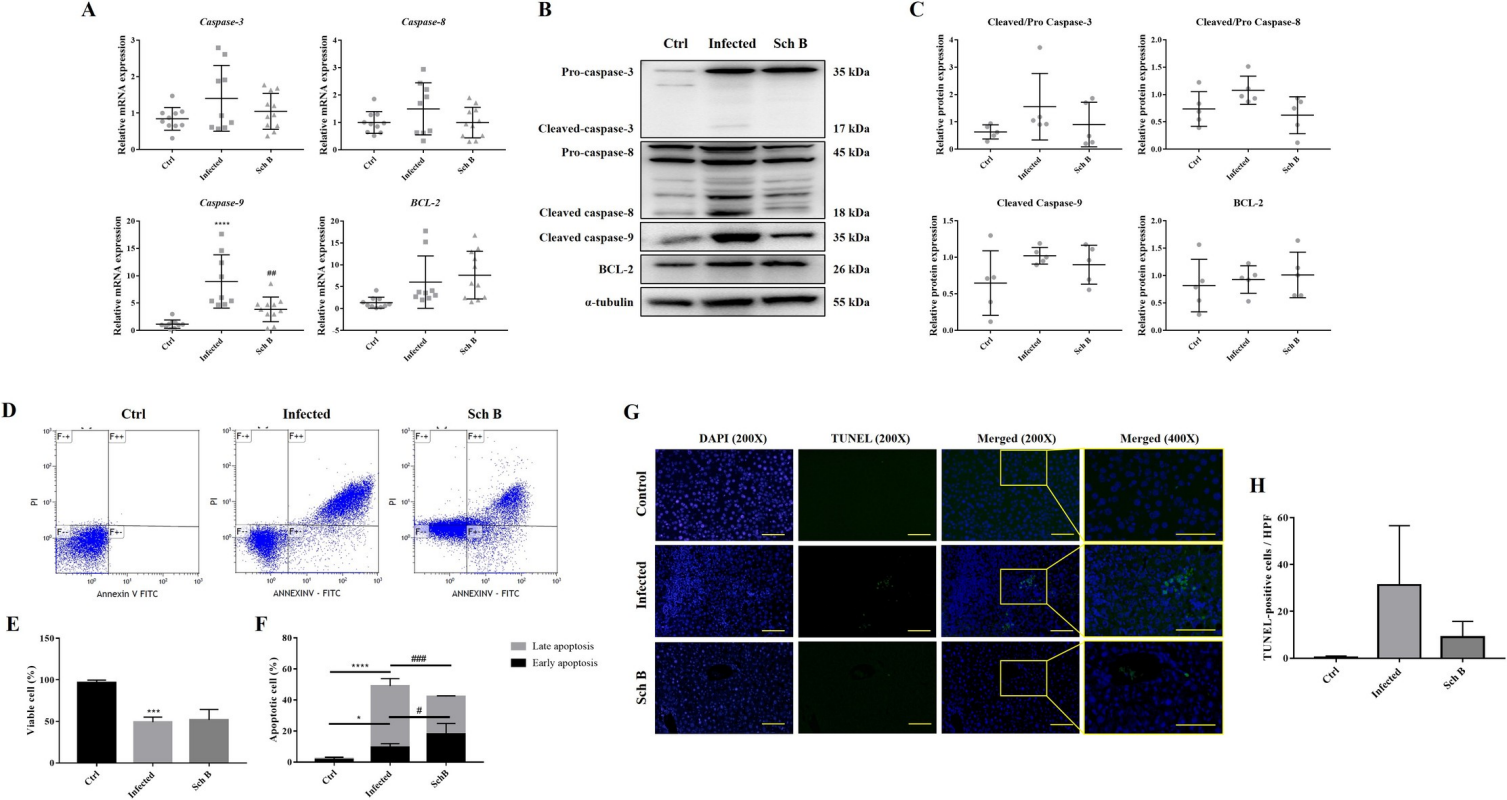
Sch B inhibits apoptosis in *S*. *mansoni*-infected mice. (A) RNA transcription levels of apoptotic markers. Data are presented as mean ± S.D. (n = 9–11). (B) Representative western blot images showing protein levels of apoptotic markers. (C) Protein expression levels of apoptotic markers, relative to that of α-tubulin. Results are presented as mean ± S.D. (n = 5). (D) Representative plots showing Annexin V-FITC and PI double staining, measured by flow cytometry. Viable cells are shown in the bottom left quadrant (double negative); early apoptotic cells are shown in the bottom right quadrant (Annexin V positive and PI negative); late apoptotic cells are shown in the upper right quadrant (double-positive). (E) Percentages of viable cells, shown as the mean ± S.D. (n = 5). (F) Percentages of apoptotic cells are shown as the mean ± SD (n = 5). (G) Representative microphotographs of TUNEL staining, shown at 200× and 400× magnifications. Scale bar corresponds to 100 μm. (H) Quantification was performed on three slides in each group. Ten random microscopic fields were counted on each slide. * *P*-value < 0.05, *** *P*-value < 0.001, and **** *P*-value < 0.0001 compared with control group; # *P*-value < 0.05, ## *P*-value < 0.01, and ### *P*-value <0.001 compared with infected group.

Apoptosis is a form of cell death which linked intimately with both physiological and pathological processes [41]. In addition to inflammation, apoptosis is also thought of as a pivotal step in the pathogenesis of liver fibrosis [42]. Here, we investigated three main apoptotic caspases including caspase-3, caspase-8, and caspase-9; and antiapoptotic protein, BCL-2. As shown in Fig 5, the expression of apoptotic caspases increased in response to *S*. *mansoni* infection whilst expression decreased following Sch B treatment (Fig 5A–5C). Expression of BCL-2 increases during infection and increase further after Sch B treatment, which is expected to happen as it inhibits the activation of apoptotic caspases (Fig 5A–5C). Flow cytometry analysis and TUNEL assay also revealed increased apoptotic cells during infection; which decreased after Sch B treatment (Fig 5D–5H).

### Schisandrin B ameliorates inflammation and apoptosis in the spleen

**Fig 6:**
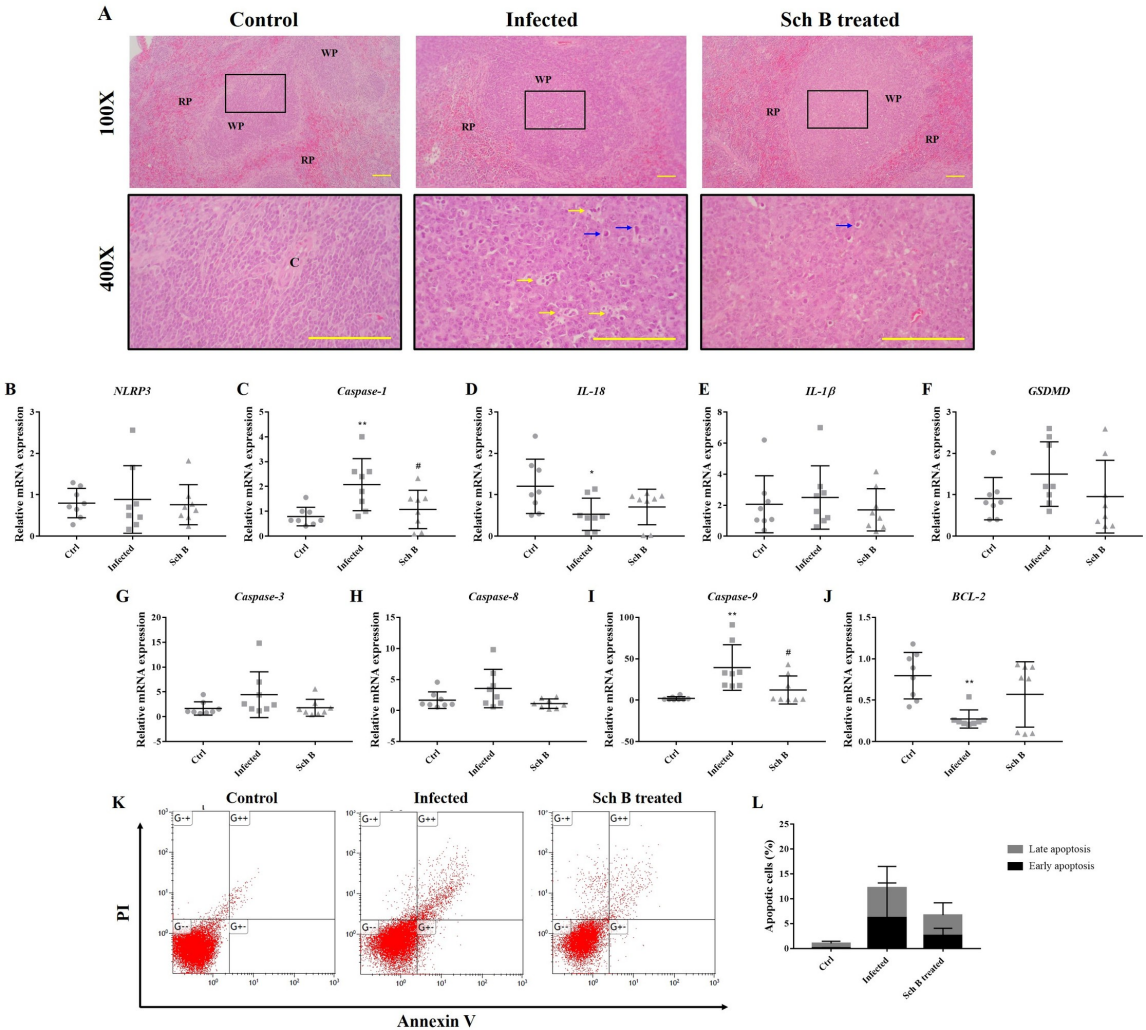
Sch B treatment resolves splenic inflammation and apoptosis in *S*. *mansoni*-infected mice. (A) Representative images showing H&E staining of spleen sections of the mice at 100× and 400× magnification. Tingible body macrophages (yellow arrows) containing apoptotic bodies (blue arrows) were seen in the splenic white pulp of *S*. *mansoni*-infected mice. Fewer apoptotic bodies were observed after *S*. *mansoni*-infected mice were treated with Sch B. Scale bars correspond to 200 μm. RP, red pulps; WP, white pulps; C, central artery. (B-F) RNA transcription levels of inflammasome markers. (G-J) RNA transcription levels of apoptotic markers. Data are presented as mean ± S.D. (n = 8). (K) Representative plots showing Annexin V-FITC and PI double staining, measured by flow cytometry. (L) Percentages of apoptotic cells are shown as the mean ± S.D. (n = 5). * *P*-value < 0.05 and ** *P*-value < 0.01 compared with control group; # *P*-value < 0.05 compared with infected group.

As we observed reduced splenomegaly and splenic size in infected mice treated with Sch B (Fig 1A and 1D), we also investigated whether Sch B has the potential in inhibiting inflammation and apoptosis in the spleen. First, we observed in the histological slides that *S*. *mansoni* infection caused significant lymphocyte apoptosis in the white pulp compartments. Apoptotic bodies phagocytized by macrophages were also seen in *S*. *mansoni*-infected mice spleen. The number of apoptotic bodies decreased after Sch B was administered to the infected mice (Fig 6A). However, no evidence of inflammation or other pathological changes were seen in the slides.

Unlike the pattern observed in the liver, only splenic *caspase-1*, *IL-1β*, and *GSDMD* increased during *S*. *mansoni* infection; whereas levels of *NLRP3* and *IL-18* did not increase during infection. Upon treatment with Sch B, levels of *caspase-1*, *IL-1β*, and *GSDMD* returned almost to the baseline as a control group (Fig 6B–6F). The unchanged level of *NLRP3* and *IL-18* suggests that although *S*. *mansoni* infection causes splenic inflammation, it was not the result of NLRP3 inflammasome activation. Regarding apoptosis, we observed an increased level of apoptotic caspases during infection, which decreased upon treatment with Sch B (Fig 6G–6I). Antiapoptotic *BCL-2* showed an opposite pattern with apoptotic caspases (Fig 6J). Flow cytometry analysis also confirmed the presence of apoptosis during *S*. *mansoni* infection; which resolves after Sch B treatment (Fig 6K and 6L). All of these results showed that Sch B has the ability to resolves inflammation and apoptosis in the spleen of *S*. *mansoni*-infected mice.

### Schisandrin B regulates immune response of *S*. *mansoni*-infected mice

Infection with *S*. *mansoni* has been shown to alters Th1 and Th2 cytokines in both mice models and humans. Decreased IFN-γ (Th1 cytokines) and increased IL-4, IL-5, and IL-10 (Th2 cytokines) during infection with *S*. *mansoni* suggested an imbalance in lymphocyte function [2,3,43,44]. Together these cytokines regulate the progression of liver fibrosis. Th1 cytokines such as IFN-γ suppress activation of hepatic stellate cells (HSCs) [45,46]; while Th2 cytokines such as IL-4 and IL-5 induce activation of HSCs [47,48]. Furthermore, the spleen takes a major part in producing cytokines after parasite infection including *Schistosoma* and liver injury [33,49–51]. To further elucidate the liver-protective effects of Sch B on *S*. *mansoni*-infected mice, cytokines concentration was measured on peripheral blood and their mRNA expression was analyzed on the mouse splenic and hepatic cells.

**Fig 7:**
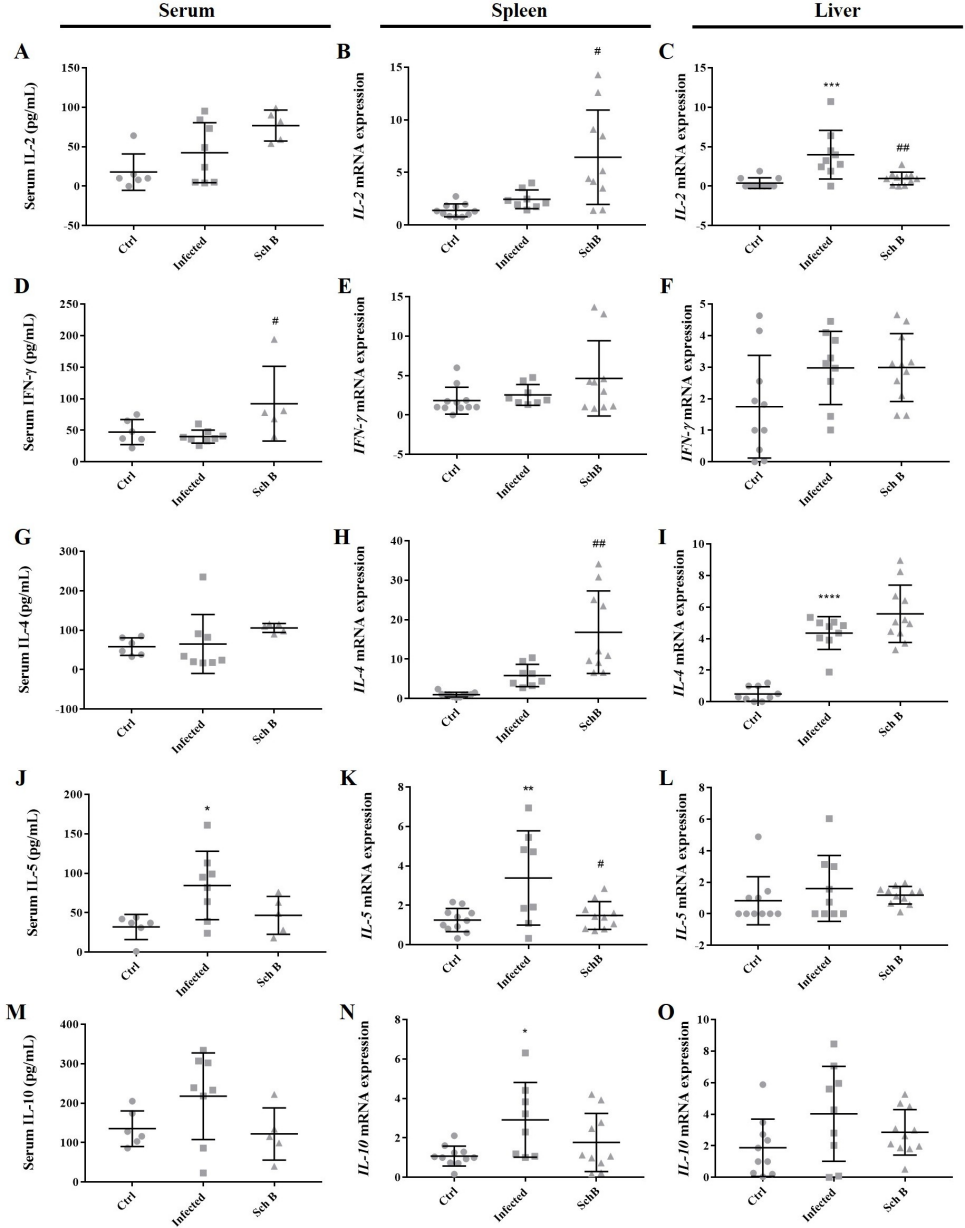
Sch B treatment regulates Th1 and Th2 immune responses in *S*. *mansoni*-infected mice. (A, D, G, J, M) Concentrations of IL-2, IFN-γ, IL-4, IL-5, and IL-10 in the serum, measured by ELISA. (B, E, H, K, N) Relative mRNA expression levels of splenic cytokines, measured by qPCR. (C, F, I, L, O) Relative mRNA expression levels of liver cytokines, measured by qPCR. IL-2 and IFN-γ are Th1 cytokines; IL-4, IL-5, and IL-10 are Th 2 cytokines. All results are presented as mean ± S.D. (n = 5–11). * *P*-value < 0.05, ** *P*-value < 0.05, *** *P*-value < 0.001, and **** *P*-value < 0.0001 compared with control group; # *P*-value < 0.05 and ## *P*-value < 0.01 compared with infected group.

As expected, mice infected with *S*. *mansoni* showed altered secretion of Th1 and Th2 cytokines. Although most of our results corroborate with previous studies, as indicated by increased Th2 cytokines levels (Fig 7G–7O), levels of Th1 cytokines (IL-2 and IFN-γ) increased unexpectedly during *S*. *mansoni* infection (Fig 7A–7F). Subsequently, a change in the cytokine expression was observed in Sch B-treated mice. Except for liver IL-2 levels, Th1 cytokines increased upon Sch B treatment (Fig 7A–7F). To be noted, Sch B treatment decreased IL-5 and IL-10 levels; while it further increased IL-4 levels (Fig 7G–7O).

### Schisandrin B causes damage on male *S*. *mansoni* adult worm

**Fig 8:**
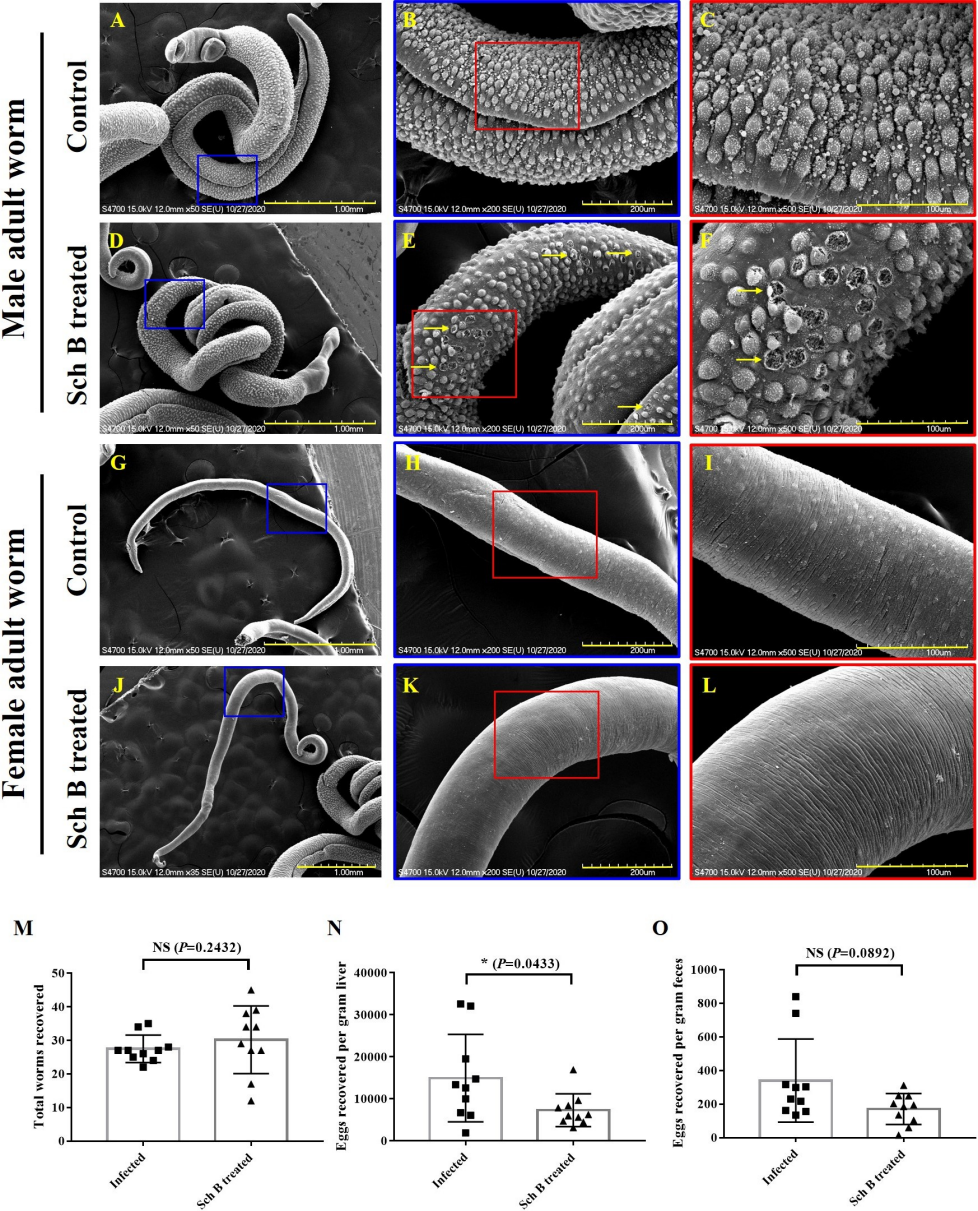
Sch B provides damage to male *S*. *mansoni*. (A-L) Representative SEM images showing the ultrastructural surface of adult *S*. *mansoni* worms isolated from the mice. (A-C) Male worms isolated from the control mice. (D-F) Male worms isolated from Sch B-treated mice. Ruptures (yellow arrows) were seen on the tubercles of the body surface. (G-I) Female worms isolated from the control mice. (J-L) Female worms isolated from Sch B-treated mice. No specific morphologic change was observed, compared with control worms. Scale bar, 1.0 mm for A, D, G, J (50× magnification, except for J: 35× magnification); 200 μm for B, E, H, K (200× magnification); 100 μm for C, F, I, L (500× magnification). (M) The number of worms recovered from the mice. (N) The number of eggs counted per gram of mouse liver. (O) The number of eggs counted per gram of mouse feces. Mann-Whitney U test was used to analyzed differences between groups. Data are presented as mean ± S.D. (n = 10). * *P*-value < 0.05 compared with control group; NS: no significance.

To explore the effect Sch B had on the *S*. *mansoni* worm, scanning electron microscopy (SEM) was performed to study the teguments of the worms. Adult worms were isolated from the mice by portal perfusion method [52]. Male *S*. *mansoni* adults isolated from control mice show ridges and tubercles all over the body (Fig 8A–8C), which are phenotypically healthy as previously described [53]. Male *S*. *mansoni* adults isolated from Sch B-treated mice showed ruptures on the tubercles and surface sloughing (Fig 8D–8F). No specific morphologic change was observed to the oral and ventral suckers (not shown). For the female adult worms isolated from the control mice, there were no presents of tubercles or spines. Generally, the female adults had a smoother surface than the males (Fig 8G–8I). However, there were no significant morphological changes observed in female adults isolated from Sch B-treated mice (Fig 8J–8L). Here we observed only mild tegmental damages on the males; whereas no damages could be observed on the females. To confirm these injuries are indeed caused by Sch B, *S*. *mansoni* adult worm pairs were incubated with different concentrations of Sch B and praziquantel (PZQ; as a positive control). Male adult worms directly exposed to Sch B showed tegument degeneration, even at the lowest concentration of 1 μM (S3A Fig); whereas no observable damage was seen on females. However, most worms remained alive upon treatment. Only at high concentrations (50 and 100 μM), the viability of the worm significantly decreased (S3B Fig). We further counted the number of worm and egg deposition in each mouse. Although Sch B treatment showed no significant change to the number of worms isolated from the mice (Fig 8M), hepatic egg deposition significantly decreased following Sch B treatment (Fig 8N). In addition, a decreased trend with *P*-value close to but not statistically significant (*P* = 0.0892) was observed in eggs recovered from stool (Fig 8O). These results also corroborated with in vitro study showing a decreased egg production when paired adult worms exposed directly to Sch B (S3C Fig).

## Discussion

Praziquantel (PZQ) is currently the only drug that has proven utility in treating human schistosomiasis. It effectively kills the worm by antagonizing the voltage-gated calcium channels of the worms [17]. However, sole treatment of PZQ cannot reverse the damages caused by the worms and prevent re-infection. In addition, the aggressive use of praziquantel has already led to drug resistance in some pandemic areas [16,18,54]. Therefore, the present study focuses on the evaluation of Schisandrin B (Sch B), a compound isolated in *S*. *chinensis*, in treating *S*. *mansoni* infection.

Improvement in the pathologic change and fibrotic markers first supports an anti-fibrotic role of Sch B in *S*. *mansoni*-induced hepatic fibrosis. Many signaling pathways are involved in liver fibrosis [55]; nevertheless, Sch B resolves liver fibrosis mainly through regulation of the TGF-β signaling [25,56]. This is also confirmed in our study, as decreased TGF-β expression was observed. Of all the fibrotic markers we investigated, fibronectin showed an unexpected trend: infection with *S*. *mansoni* only slightly increased its expression but treatment with Sch B largely increased its expression levels (Fig 2A and 2J). Fibronectin is essential in maintaining the composition and stability of extracellular matrix (ECM) and supports a wide variety of cellular functions such as cell adhesion, proliferation, and differentiation [57]. Since excessive fibronectin leads to an impairment of tissue structures and the development of fibrotic tissues [58,59], it is supposed that the fibronectin expression will be increased in our *S*. *mansoni*-infected mice model. However, our findings here did not corroborate previous findings that show increased fibronectin in egg-induced granulomas [60,61]. One previous study may explain this: a low level of hepatic fibronectin increases collagen levels and leads to more severe hepatic fibrosis [62]. Also, in the same study, the authors found that pathogenesis of fibrosis in liver lacking fibronectin is the result of activated-TGF-β [62], which corroborates our findings with increased collagen I and TGF-β levels (Fig 2D, 2B, 2K and 2M). We also postulate that the upregulated levels of fibronectin in Sch B treated mice are to protect against exacerbation of fibrosis [62].

As fibrosis is usually related to inflammation and apoptosis [36,42,63], we also investigated some key effectors of it. The results proved that Sch B played a vital role in downregulating inflammasome activation in macrophages and protected the liver from apoptosis (Figs 3–5). *S*. *chinensis*, the plant where Sch B was isolated, has long been confirmed to exert an anti-inflammatory property [64] and has been used widely in traditional Chinese medicine practices. Numerous reports have shown that Sch B suppresses inflammasome activation to achieve a protective effect against different disease model [33,65–67]. Additionally, Sch B has been shown to inhibit inflammasome activation and pyroptosis in both peritoneal macrophages and RAW264.7 cells [66], which is similar to our findings in this study. The inhibitory effects of Sch B on inflammasome may involve regulation of Nrf2/NF-κB or MAPK/ NF-κB signaling, as most of the studies showed [30,68–71]. This, however, is yet another area of inquiry for future investigation. Also, alleviation of inflammation in our study was accompanied by decreased apoptosis (Fig 5). Apoptosis occurs as a proinflammatory process during a pathological condition [72]. During pathological apoptosis, apoptotic cell fragments excessively accumulated in the tissues and release their inflammatory contents. In addition, activated Kupffer cells in the liver secretes inflammatory TNF-α and heightens the expression of Fas ligand, therefore expediting liver cell inflammation and apoptosis [73]. Studies have also confirmed the activation of apoptosis by different inflammasomes, which is parallel to caspase-1-induced pyroptosis [9,10]. Although Sch B has proven beneficial to inhibit inflammatory and apoptotic proteins in the liver of *S*. *mansoni*-infected mice, only little benefit was observed in increasing the number of viable cells (Fig 5D and 5E). A possible reason might be that Sch B could not clear the trapped eggs in the liver (Figs 1G and 8N) so that the trapped eggs persisted to damage the hepatocytes. To also exclude the possibility that apoptosis originated from egg-targeting immune and inflammatory cells, we performed TUNEL staining to the liver section of the mice. We counted only the TUNEL-positive hepatocytes and the results confirmed that Sch B can lessen liver cells apoptosis (Fig 5G and 5H).

We also demonstrated that Sch B is efficacious in alleviating inflammation and apoptosis in the spleen, as confirmed by histology analysis and their mRNA expression (Fig 6A). Despite *S*. *mansoni* infection largely induces NLRP3 activation in the liver, it appears that NLRP3 only slightly activates in the spleen (Fig 6B). In the meantime, the inflammasome effectors including caspase-1, IL-1β, and GSDMD largely increased at infection; whereas IL-18 decreased during infection (Fig 6C–6F). Similarly, all of these inflammasome-related markers decreased in expression following Sch B treatment; except for IL-18, which showed an increased expression. One study has confirmed the increase of NLRP3 in the spleen of *S*. *japonicum*-infected mice [40], but none has shown the effect of *Schistosoma* infection on the downstream effector proteins. Hence, we conceivably hypothesized that there might be other pathways involved in the change of IL-18.

Immune response has shown a crucial role in *Schistosoma* infection. Decreased Th1 and increased Th2 response contribute to the development of liver fibrosis in schistosomiasis [2,3,43]. Interestingly, in our study, levels of IL-2 (a Th1 cytokine) increased following *S*. *mansoni* infection (Fig 7A and 7B). This seems to be in contrast with previous studies. It has been shown that following the development of liver fibrosis (starting from around six-week post-infection), the immune response strongly polarized to Th2, which is induced by the egg antigen [3,44]. At that time, stimulated-liver tissue or isolated granulomas from infected mice failed to produce IL-2 [44]. However, one study suggested that administration of IL-2 enhances the severity of fibrosis development in the schistosomiasis model [74]. In the current study, although we could not clarify the origin of increased IL-2, we believe the increase of IL-2 is to worsen the immunopathology of the liver, as our other results showed (Figs 1 and 2). The use of Sch B reversed the immune response from Th2 to Th1, as shown by increased IL-2 and IFN-γ; and decreased IL-5 and IL-10. Notwithstanding, IL-4 (a Th2 cytokine) increased following Sch B treatment (Fig 7E and 7F). In addition to its pro-fibrotic properties, IL-4 is also an anti-inflammatory cytokine [75,76]. We therefore suggested an anti-inflammatory role of IL-4 following Sch B treatment, which correlated with reduced liver inflammation (Fig 3).

Lastly, we looked into the effect Sch B has on the worm. Although Sch B causes slight damage to the male adult worm (Fig 8D–8F), it does not alter the number of total worms (Fig 8M) or male-to-female worm ratio (S2 Fig) in the mouse. This is similar to in vitro study showing that direct exposure to Sch B causes damage on male worms (S3A Fig) but did not kill the worms (S3B Fig). However, the number of hepatic eggs and feces eggs decreased following Sch B treatment (Fig 8N and 8O), as Sch B affects the reproductive process of the worms (S3C Fig). Differences in the damage to different sex could be explained by their mating position. During mating, female worms reside in the gynecophoral canal of male worms for sexual maturation and egg production [77,78], making the female less likely to be exposed to Sch B. Male worms are therefore exposed and become irreparably damaged. Sexual maturation is the prerequisite of producing eggs [79]. In order to be sexually mature, female-male contact is indispensable [78,80,81]. However, male worm damaged by Sch B may reduce contact with female worm or make the contact less sufficient. This helps reduces egg output and consequently parasite burden and disease progression.

Although some study suggested that the persistent use of PZQ may also provide an anti-fibrotic effect against schistosomiasis [82–84], this beneficial effect usually occurs at an earlier stage [84–86]. PZQ treatment decreases the number of hepatic eggs and therefore leads to a decrease in granuloma and fibrotic tissue formation. However, PZQ has only a little or no effect on the already established fibrotic tissues and is therefore not beneficial for patients in the later stage of schistosomiasis [84,86]. Sch B, in addition to the anti-parasitic effects shown in this study, can also reverse the existing fibrosis [25,56]. Sch B treatment also provided better resolution of liver fibrosis than PZQ treatment (S4 Fig). A longer duration of Sch B treatment or combination treatment with PZQ may lead to a better treatment outcome, and further investigation is required to confirm this hypothesis.

In conclusion, the present study demonstrates that Sch B is beneficial in treating *S*. *mansoni*-induced liver fibrosis and splenic damages, through inhibition of inflammasome activation and apoptosis; and immune regulation. In addition, Sch B causes damages to the male worms, therefore helps to reduce egg production and lessen the parasite burden.

## Materials and methods

### Ethics statement

All procedures involving animals were approved by the Institutional Animal Care and Use Committees (IACUC) of Tzu Chi University (No. 109066) and were carried out under approved guidelines of the National Institutes of Health (NIH) Guide for the Care and Use of Laboratory Animals (DHHS publication No. NIH 85–23, revised 1996).

### Parasites and animals

Puerto Rico strain of *Schistosoma mansoni* (*S*. *mansoni*) was obtained from the Biomedical Research Institute, Rockville, MD 20852, USA, and was maintained in our laboratory. The freshwater snail *Biomphalaria glabrata* was used as an intermediate host and eight-week-old male BABL/c mice (National Laboratory Animal Center, Taipei, Taiwan) were used as the final host. BALB/c mice were housed under a 25°C ± 2°C and a 12 h light/dark cycle condition with free access to water and food.

### Animal treatment

Mice were divided into three groups—one control (n = 15), one infection (n = 24), and one treatment (n = 26) group. Each mouse of the infection and treatment group was percutaneously infected with 100 ± 20 cercariae via the tail, and the control group was treated with normal saline. The treatment group was further treated with 20 mg/kg/day Sch B (Chengdu Alfa Biotechnology, Chengdu City, Sichuan, China) by oral gavage for 28 consecutive days, starting from week four post-infection. All groups were euthanized at week eight post-infection. Ten mice from the infection and treatment group were used for worm reduction analysis. The rest of the mice were dissected and examined for any pathological changes in the liver and spleen. Five liver specimens were obtained for western blotting and histology; while the rest were used for qPCR and flow cytometry.

### Histopathological examination

Tissues were harvest and immediately fixed with 10% formalin. After fixation, tissues were dehydrated in a series of graded dilutions of alcohols: 70% (2 × 30 min), 95% (2 × 30 min), and 100% (2 × 30 min). The procedures were followed by immersion in xylene (2 × 30 min) and a paraffin bath (30 min). Tissues were then put in an embedding cassette with melted paraffin. Paraffin-embedded tissues were then sectioned into thin slices using a microtome. Before proceeding with the staining protocol, the slides were deparaffinized and washed with Sub-X (Leica Biosystems, Richmond, IL, USA), 100%, 95%, 75%, and 50% ethanol. For hematoxylin & eosin (H&E) staining, the rehydrated slides were stained as follows: hematoxylin solution (Merck, Darmstadt, Germany; 3 min), tap water (1 min), 70% ethanol with 1% HCl (5 s), tap water (1 min), eosin solution (3 min), 95% ethanol, 100% ethanol, and Sub-X. Masson’s trichome staining was performed by trichrome stain kit (ScyTek Laboratories, Inc., West Logan, USA). Slides were incubated with pre-heated Bouin’s fluid for 60 min and then stained as follows: Weigert’s solution (10 min), biebrich scarlet/acid fuchsin solution (15 min), phosphomolybdic/phosphotungstic acid solution (15 min), 1% aniline blue solution (5 min), 95% ethanol, 100% ethanol, and Sub-X. Sirius red staining was performed using a picro-sirius red stain kit (ScyTek Laboratories, Inc., USA). The slides were stained with picro-sirius red solution (60 min), 0.5% acetic acid (2 × 5 s), 100% ethanol, and Sub-X. The severity of liver fibrosis was scored according to the Ishak fibrosis scoring system as previously described [87,88].

### Serum biochemical analysis

Whole blood was obtained by cardiac puncture and was centrifuged at 600× g for 10 min to obtain the serum. Serum was then analyzed for alanine transaminase (ALT), aspartate transaminase (AST), and lactate dehydrogenase (LDH) using a Hitachi 7080 Chemistry Analyzer (Hitachi Ltd., Tokyo, Japan).

### RNA isolation, cDNA synthesis, and quantitative real-time PCR analysis

Total RNA was isolated using TRIzol reagent (Thermo Scientific, Rockford, IL, USA), according to the manufacturer’s instructions. 5 μg of RNA was used for cDNA synthesis using RevertAid first strand cDNA synthesis kit (Fermentas International Inc., Burlington, ON, Canada). The synthesized cDNA was used for qPCR experiments. qPCR was performed with the LabStar SYBR qPCR kit (Bioline, London, UK) using the Roche LightCycler 480 System. The primers used in this study are shown in the S1 Table. Relative gene expression was calculated using the ΔΔCt method and gene expression levels were normalized to *β-actin*.

### Western blot analysis

Protein samples were separated by 8%, 10%, or 12% sodium dodecyl sulfate-polyacrylamide gels (SDS-PAGE) and then transferred onto PVDF membranes (EMD Millipore, Burlington, MA, USA). After being blocked with 5% non-fat milk, the membranes were incubated with the following primary antibodies at 4°C overnight: α-tubulin (GeneTex, Irvine, CA, USA), collagen I (GeneTex), α-SMA (Proteintech, Chicago, IL, USA), TGF-β (Cell signaling technology, Danvers, MA, USA), TNF-α (GeneTex), NLRP3 (Proteintech), caspase-1 (Proteintech), IL-18 (Proteintech), IL-1β (Proteintech), caspase-3 (GeneTex), caspase-8 (GeneTex), caspase-9 (GeneTex), and BCL-2 (GeneTex). Membranes were then incubated with HRP-conjugated mouse anti-IgG (EMD Millipore) or HRP-conjugated rabbit anti-IgG (EMD Millipore) secondary antibodies for 1 h. Membranes were developed using ECL detection reagent (EMD Millipore). Relative protein levels were quantified using Image J (Version 1.46, National Institute of Health, Bethesda, MD, USA), and protein densitometry were expressed relative to that of α-tubulin.

### Assessment of MMP-2 and MMP-9 by gelatin zymography

80 μg/mL tissue protein were loaded on 8% SDS-PAGE that had been co-polymerized with 0.1% gelatin. Electrophoresis was performed at 120 V for 1 h. The gels were then washed two times for 30 min in washing buffer (2.5% Triton X-100, 50 mM Tris-HCl, pH 7.5, 5 mM CaCl_2_, 1 μM ZnCl_2_). After washed with distilled water, the gels were incubated in an incubation buffer (1% Triton X-100, 50 mM Tris-HCl, pH 7.5, 5 mM CaCl_2_, 1 μM ZnCl_2_) for 24 h at 37°C. The gels were at last stained with Coomassie brilliant blue for 1 h, and de-stained in 15% methanol/7.5% acetic acid. The activity of MMP-2 and MMP-9 was detected as a clear band against a blue background.

### Immunohistochemistry staining

Paraffin slides were deparaffinized and rehydrated. Antigens were retrieved from the sections by soaking them in boiling EDTA buffer for 20 min. Subsequently, the sections were treated with 3% H_2_O_2_ for 10 min. Thereafter, the sections were incubated overnight at 4°C with the following primary antibodies: MPO (ABclonal), CD45 (ABclonal), and CD68 (ABclonal). The sections were then incubated with HRP-conjugated secondary antibody (EMD Millipore) for 30 min and 3, 3′-diaminobenzidine (DAB; Thermo Scientific) for 3 min. Sections were counterstained with Hematoxylin (Merck) and rehydrated with increasing concentration of ethanol.

### Cell culture and proliferation assay

RAW 264.7 cells were cultured with Roswell Park Memorial Institute (RPMI) 1640 medium (Gibco; Thermo Fisher Scientific, Inc.) supplemented with 2 mM L-glutamine, 1% penicillin-streptomycin (Biowest, MO, USA), and 10% fetal bovine serum (FBS; Gibco; Thermo Fisher Scientific, Inc.); and cells were maintained in a 5% CO_2_ humidified incubator at 37°C. For proliferation assay, cells were seeded at 8,000 cells per well in 96-well plates. After incubating overnight, cells were treated with 0.1 or 1.0 μg/mL SEA with or without 10 or 25 μM Sch B. For the control group, cells were treated with an equal volume of medium. Following incubation of 24 or 48 h, the medium was aspirated, and 100 μL WST-1 (1:10 dilution in culture medium) solution (Roche, Indianapolis, IN, USA) was added and incubated for 30 min at 37°C. The absorbance was measured at a wavelength of 450 nm.

### Preparation of *S*. *mansoni* soluble egg antigen (SEA)

Extra mice infected with *S*. *mansoni* were sacrificed at eight weeks post-infection. Livers were collected and were homogenized in ice-cold 0.85% NaCl. Eggs were separated from liver tissue by passing the tissue through a series of sieves with decreasing pore size: 420, 177, 105, and 25 μm. The eggs retained on the 25 μm sieve were collected in ice-cold PBS, followed by centrifugation at 370× g for 2 min. The centrifuged eggs were then resuspended in ice-cold PBS. Soluble egg antigen (SEA) was prepared by homogenizing the eggs with a glass homogenizer.

### Preparation of mouse liver and splenic cells

Cell suspension from the mouse liver and spleen were prepared by dispersing the tissue in 8 mL phosphate-buffered saline (PBS) containing 5% FBS using a syringe needle. 8 mL red blood cell (RBC) lysis buffer (0.15 M NH_4_Cl, 1 mM KHCO_3_, 0.1 mM Na_2_EDTA; pH 7.2–7.4) were added to lysed the erythrocytes. After centrifugation at 250× g for 5 min, cells were resuspended in 5% FBS-containing PBS and were passed through a 70 μm nylon mesh cell strainer (Corning, Inc., Corning, NY, USA) [89,90].

### Determination of apoptosis using flow cytometry

Apoptotic cell death was measured by Alexa Fluor Annexin V/Dead Cell Apoptosis kit (Molecular Probes Inc., Eugene, OR, USA). Cells were washed and resuspended in ice-cold binding buffer and stained with 5 μL Annexin V-FITC and PI in the dark for 15 min. Thereafter, 400 μL binding buffer was added, and the cells were analyzed using a Gallios 10-channel flow cytometer (Beckman Coulter, Brea, CA, USA).

### Terminal deoxynucleotidyl transferase dUTP nick end labeling (TUNEL) assay

TUNEL staining was performed using Andy Fluor 594 apoptosis detection kit (GeneCopoeia, Rockville, USA), according to the manufacturer’s instructions. Briefly, the slides were deparaffinized, rehydrated, and rinsed with distilled water. Pretreatment was carried out using proteinase K. The slides were then treated with TdT reaction buffer for 10 min, followed by TdT reaction cocktail for 2 h at 37°C. After washing, the slides were incubated with streptavidin staining solution for 30 min. Finally, the slides were washed and mounted.

### Worm burden, stool, and hepatic eggs count

Adult worms were isolated by portal perfusion method [52]. The number of worms was collected and counted under a dissecting microscope. Worm burden reduction was calculated as:

$$\frac{Worm\;recovered\;from\;infected\;group-Worm\;recovered\;from\;treatment\;group}{Worm\;recovered\;from\;infected\;group}\times 100\%$$

Stool and hepatic eggs were counted using the Kato-Katz technique [91] directly performed on stool or liver tissues. Egg burdens were expressed as eggs per gram of stool or liver. The egg reduction rate was calculated as:

$$\frac{Eggs\;counted\;from\;infected\;group-Eggs\;counted\;from\;treatment\;group}{Eggs\;counted\;from\;infected\;group}\times 100\%$$

### Cytokine ELISA

Concentrations of IL-4, IL-10, IL-2, IL-5, and IFN-γ in the sera were measured using a standard sandwich ELISA kit (Thermo Fisher Scientific, Waltham, MA, USA) following the manufacturer’s instructions. Briefly, 96-well ELISA plates were coated overnight at 4°C with 100 μL capture antibody. On the next day, the capture antibody was discarded, wells were washed, and 200 μL ELISA/ELISASPOT diluent was added. After 1 h incubation, the wells were washed and incubated with 100 μL samples or standards for 2 h. Then, plates were washed and 100 μL detection antibody was added and incubated for 1 h. The wells were then washed and incubated with 100 μL Avidin-HRP enzyme for 30 min. After washing, 100 μL 3,3’,5,5’-tetramethylbenzidine (TMB) substrate was added and incubated for 15 min. 10% sulfuric acid was applied to stop the reaction, and the optical density was determined at 450 nm.

### Scanning electron microscopy (SEM)

Adult worms from the mice were isolated from the portal vein and mesenteric veins by portal perfusion method [52]. After washing with PBS, the worms were fixed in 2.5% glutaraldehyde for 60 min at 4°C. Worms were then rinsed twice with 5% sucrose. After that worms were incubated with 1% osmium tetroxide for 60 min, increasing concentrations of ethanol (50%, 70%, 80%, 90%, and 100%; 10 min each) were used to dehydrate the worms. Worms were critically point-dried and sputter-coated with gold. A HITACHI S-4700 field emission scanning electron microscope (Hitachi Ltd, Tokyo, Japan) was used to visualize the worms.

### Parasite’s culture and drug treatment

Adult *S*. *mansoni* were isolated from infected mice by portal perfusion method [52] with pre-warmed Dulbecco’s Modified Eagle Medium (DMEM; Gibco; Thermo Fisher Scientific, Inc., Waltham, MA, USA) supplemented with 5% FBS and sodium citrate. Worms were washed several times before putting into the culture medium (DMEM supplemented with 1% penicillin-streptomycin and 10% fetal bovine serum). To investigate the effect of Sch B or PZQ on the worms, three *S*. *mansoni* adult worm pairs were placed in 24-well plates with 1 mL culture medium supplemented with indicated concentrations of Sch B or praziquantel (PZQ; as positive control). The worms were observed every day under a dissecting microscope for their morphological appearance and viability. For suspicious worms, stimulation with a plastic needle was done to determine their “alive or dead” status as previously described [92]. The viability of the worm was scored as 0, dead; 1, immobile but respond to needle stimulation; and 2, alive with mobility. To also investigate the reproductive process, medium was changed every 24 h to count the number of eggs [92,93]. All plates were incubated at 37°C in a humidified atmosphere of 5% CO_2_. All experiments were performed in triplicates.

### Statistical analysis

All experimental data were analyzed using GraphPad Prism 6.01 software (GraphPad Software Inc., San Diego, CA, USA). Data are represented as the mean ± standard deviation (S.D) unless stated otherwise. For most of the study, one-way analysis of variance (ANOVA) was used, followed by a Tukey’s post-hoc test to determine differences between groups. For worm burden, stool, and hepatic egg count analysis, Mann-Whitney U test was used to compare differences between the infection and Sch B treated group. A *P*-value < 0.05 indicates a significant difference.

## Supporting information

S1 Fig Immunohistochemistry staining of immune cell markers. Representative liver sections stained with different immune cell markers including CD68 (macrophages), MPO (neutrophils), and CD45 (leukocytes). Yellow arrows suggested clusters of immuno-positive cells. Histograms represent mean ± S.D from three slides in each group. Images are shown at 400× magnifications and scale bars correspond to 200 μm. * *P*-value < 0.05 and ** *P*-value < 0.01 compared with control group; # *P*-value < 0.05 compared with infected group.
(TIF)

S2 Fig The number of male and female worms isolated. No statistical difference was seen in the number between male and female worms. Mann-Whitney U test was used to analyzed differences between groups. Data are presented as mean ± S.D. (n = 10). NS, no significance.
(TIF)

S3 Fig Sch B causes damage to male worms and affects reproduction of paired *S*. *mansoni* worms in vitro. (A) Representative images showing morphological changes in male worms cultured in media containing different concentrations of Sch B and PZQ (as a positive control) at day 7. Control, worms cultured in media alone. Tegument degeneration (yellow arrows) was seen on male adult worms. (B) Worm viability score and bar chart showing area under curve (AUC). (C) In vitro production of eggs and bar chart showing area under curve (AUC). All results are presented as the mean ± S.E.M from three independent experiments. ** *P*-value < 0.01, *** *P*-value < 0.001, and **** *P*-value < 0.0001 compared with control group.
(TIF)

S4 Fig Sch B treatment provides a better resolution of liver fibrosis than PZQ treatment. (A) ALT and (B) AST levels measured in the serum. Results are shown as mean ± S.D. (n = 3). (C) Representative images showing H&E staining of liver sections of the mice. Fibrotic areas were encircled by a yellow dotted line. (D) Fibrosis was evaluated by Ishak fibrosis scoring based on H&E staining. (E) Representative images of Masson’s trichrome staining on liver sections of the mice. Collagen were stained as blue which was encircled by a yellow dotted line. (F) Ishak fibrosis scoring based on Masson’s trichrome staining. For quantification in (C and E), the number and the corresponding areas of each circled area were potted directly on the graph. Each dot represents one fibrotic or positively stained area. Quantification or scoring was performed on three slides in each group. 15 random microscopic fields were examined on each slide. Images are shown at 100× magnifications and scale bars correspond to 200 μm. * *P*-value < 0.05, ** *P*-value < 0.01, *** *P*-value < 0.001, and **** *P*-value < 0.0001 compared with control group; # *P*-value < 0.05 and # *P*-value < 0.01 compared with infected group.
(TIF)

S1 Table Primer pairs used in this study. (XLSX)
