# Supplementary figures and images for: Ameliorative effects of Schisandrin B on Schistosoma mansoni-induced hepatic fibrosis in vivo
Source: PLoS Negl Trop Dis. 2021 Jun 23;15(6):e0009554. doi: 10.1371/journal.pntd.0009554 (PMC8259995; doi:10.1371/journal.pntd.0009554)

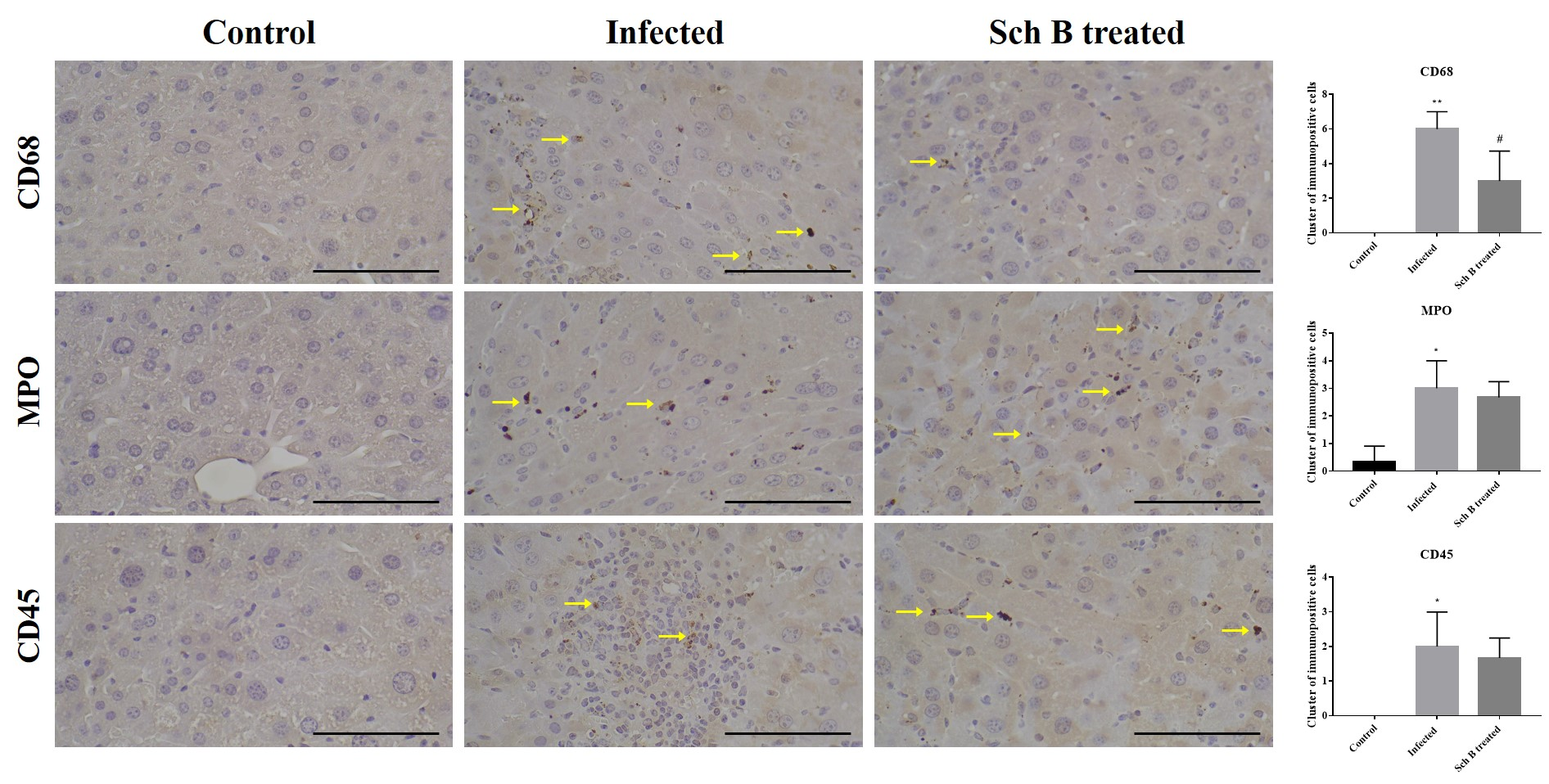

Supplement: S1 Fig — Representative liver sections stained with different immune cell markers including CD68 (macrophages), MPO (neutrophils), and CD45 (leukocytes). Yellow arrows suggested clusters of immuno-positive cells. Histograms represent mean ± S.D from three slides in each group. Images are shown at 400× magnifications and scale bars correspond to 200 μm. * P-value < 0.05 and ** P-value < 0.01 compared with control group; # P-value < 0.05 compared with infected group. (TIF) [file pntd.0009554.s001.tif]

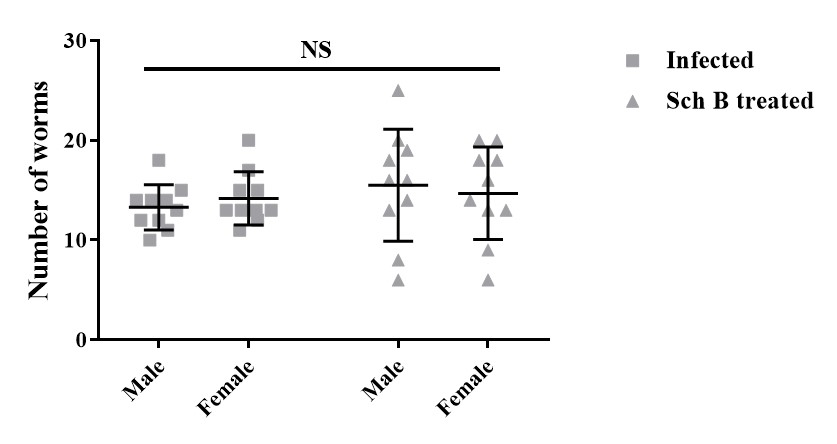

Supplement: S2 Fig — No statistical difference was seen in the number between male and female worms. Mann-Whitney U test was used to analyzed differences between groups. Data are presented as mean ± S.D. (n = 10). NS, no significance. (TIF) [file pntd.0009554.s002.tif]

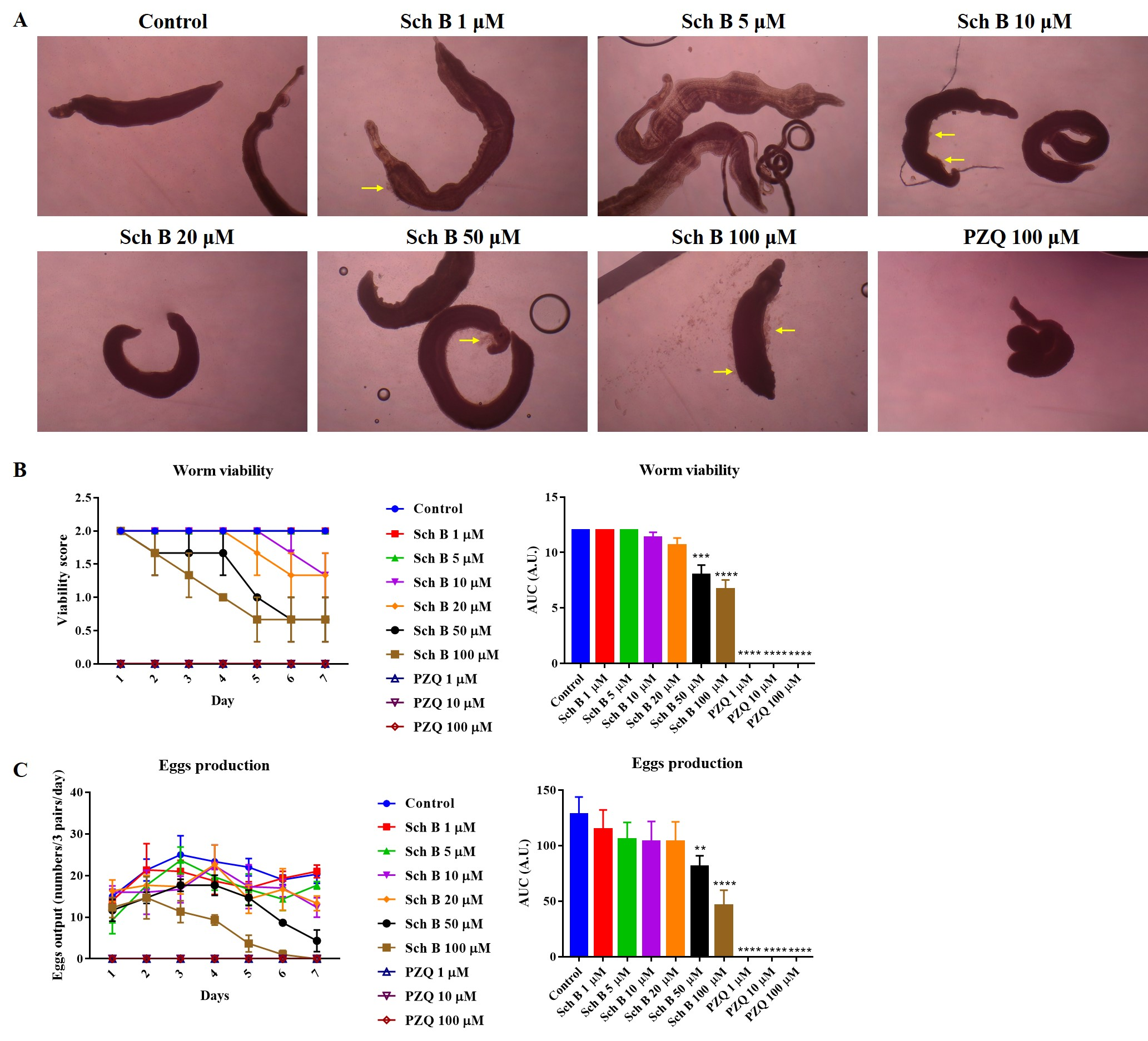

Supplement: S3 Fig — (A) Representative images showing morphological changes in male worms cultured in media containing different concentrations of Sch B and PZQ (as a positive control) at day 7. Control, worms cultured in media alone. Tegument degeneration (yellow arrows) was seen on male adult worms. (B) Worm viability score and bar chart showing area under curve (AUC). (C) In vitro production of eggs and bar chart showing area under curve (AUC). All results are presented as the mean ± S.E.M from three independent experiments. ** P-value < 0.01, *** P-value < 0.001, and **** P-value < 0.0001 compared with control group. (TIF) [file pntd.0009554.s003.tif]

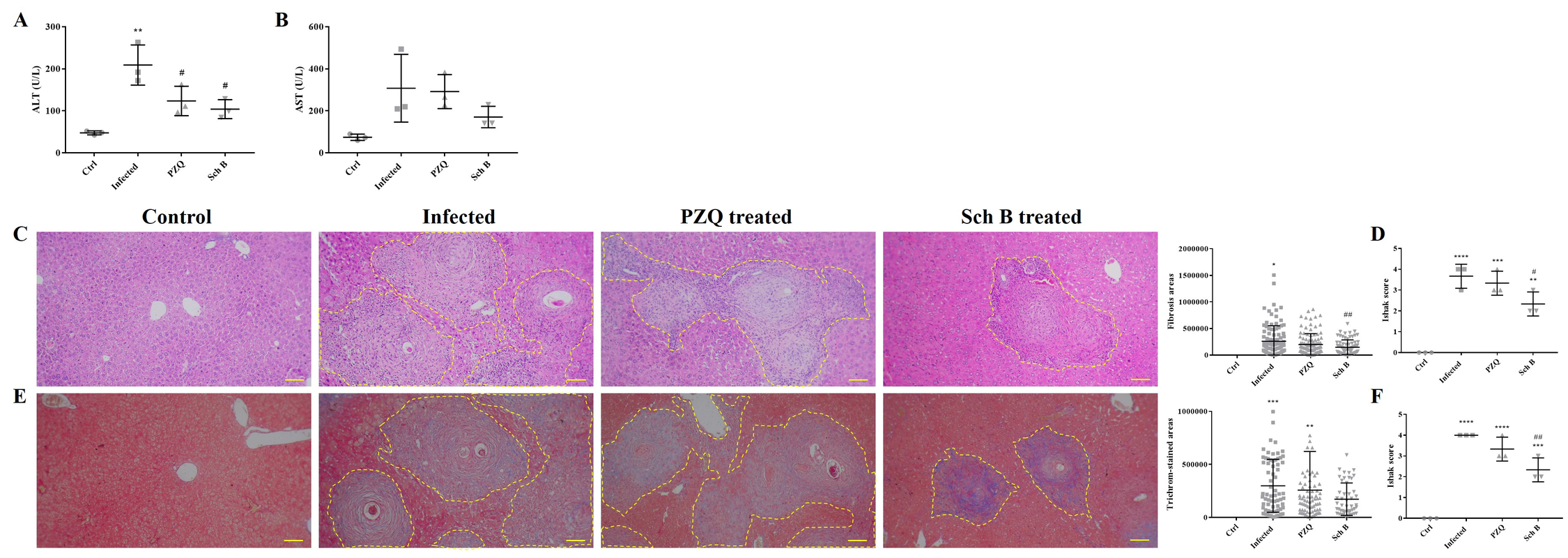

Supplement: S4 Fig — (A) ALT and (B) AST levels measured in the serum. Results are shown as mean ± S.D. (n = 3). (C) Representative images showing H&E staining of liver sections of the mice. Fibrotic areas were encircled by a yellow dotted line. (D) Fibrosis was evaluated by Ishak fibrosis scoring based on H&E staining. (E) Representative images of Masson’s trichrome staining on liver sections of the mice. Collagen were stained as blue which was encircled by a yellow dotted line. (F) Ishak fibrosis scoring based on Masson’s trichrome staining. For quantification in (C and E), the number and the corresponding areas of each circled area were potted directly on the graph. Each dot represents one fibrotic or positively stained area. Quantification or scoring was performed on three slides in each group. 15 random microscopic fields were examined on each slide. Images are shown at 100× magnifications and scale bars correspond to 200 μm. * P-value < 0.05, ** P-value < 0.01, *** P-value < 0.001, and **** P-value < 0.0001 compared with control group; # P-value < 0.05 and # P-value < 0.01 compared with infected group. (TIF) [file pntd.0009554.s004.tif]
